# Supplementary material for: Patterns of Intron Gain and Loss in Fungi
Source: PLoS Biol. 2004 Nov 30;2(12):e422. doi: 10.1371/journal.pbio.0020422 (PMC532390; doi:10.1371/journal.pbio.0020422)
Supplement: Table S1 — Also available at http://genes.mit.edu/NielsenEtAl/. (4.3 MB ZIP). [file pbio.0020422.st001.zip › NielsenEtAl/html/617.html]

AN7428.1.NCU06672.1.MG07297.1.FG10226.1


```
 CLUSTAL W (1.82) Multiple Sequence Alignments - Introns Inserted


Sequence 1: NCU06672.1	699 aa
Sequence 2: FG10226.1	694 aa
Sequence 3: MG07297.1	718 aa
Sequence 4: AN7428.1	617 aa
Alignment Length: 729 aa
Number Identitical Residues: 299 aa
Alignment Score (without introns) 15820


MG07297.1 	MSGNDEAAAAGVAPPQTLQFAPFESQIEMPFYSALFSRKLDHDKLDDSVRPVIGLYQPMS
NCU06672.1	---------------MDLKFATFSSEIELPFYSALFSSKLDHDKLDSSARPVLGLYEPRS
FG10226.1 	-------------MAKPLQFAPFISEIELPFYSALFASKLDHDKLDDSARSVLGLYEPRS
AN7428.1  	-----------------MQYTPFASDIELPFYIALASLKINHDKLDDSARKVLGLYELRP
          	                 ::::.* *:**:*** ** : *::*****.*.* *:***:  .

MG07297.1 	ERPPAESTRMQIQGGALSSS-~Q2P~IVS~VPMGYTRADGSIRNFNTIEDFKKADKGAIL
NCU06672.1	HASPEASTRMQILGSALTS--~D~Q2DES~GPLGMTRAEGYIKNVNTIEEFKNTDKNAMI
FG10226.1 	EE-PESSCRLQILGNALTSGK2T~N~EPS~SPLATMRAEGIIRNVNTLEDFKNTDKPAML
AN7428.1  	SDAPNASCRIQIHGNALTS--~-~-~-DE2VPSTYYRAEGMIKNVNTIEEYAKADKMGML
          	  .*  * *:** *.**:*         .  *    **:* *:*.**:*:: ::** .::

MG07297.1 	RQAGAQ0IWDAIKDGSIYEIPSLLSSFAILSYADLKKYRFTYWFAYPTLHSVPAWR----
NCU06672.1	KKAGEQ0IWDAIQDGTIYSCPSLLASFRILSYADLKKYKFTYWFAFPALHSEPQWK----
FG10226.1 	RTAGRQ0VWDAIKDGSIYSVPSLLSSFIILSYADLKKYKFTYWFAFPALHSDPVWK----
AN7428.1  	QQSGET0IWNAINNGTIYSCPSLLSAFVILSYADLKKYKFHYWFAFPALHSDPSWTPLEE
          	: :*   :*:**::*:**. ****::* **********:* ****:*:*** * * . ..

MG07297.1 	--RDGPLARFSSKETTALVNEVGTFRYAHDTRQHGFFLAKKVPYRSGPFRRGLPRDDSDG
NCU06672.1	--RTGPIGRLTSDESTALVERIGTWRYMVDRREHGFFLAKKV--RREAAGPRSSLDDPGV
FG10226.1 	--RSGPAERLTSQETTALVDRVGTWRYSVDARERGFFLAKKVPGRRETDDP-----DTPQ
AN7428.1  	GCEGAQAHRLPSVESSALARSVQEWARVVDAPQRGFFLARRVRMRDDDTVS---------
          	... .   *:.* *::**.  :  :    *  ::*****::*  *               

MG07297.1 	DDIGFTWSIGALGEFEKGFFKGIKEEDQYIAFVDSSSYAENPSWPLRNLLVLIRQRFQLQ
NCU06672.1	D-IGYRWDIGSLRDFETGFFNDAAEEDRYVAFVDPSNYPEYPSWPLRNLLILIRQRYRLN
FG10226.1 	E-LPFHWEIGSLRDFETGFFDQVPEEDRYVAFTDPSTYPEAPGWPLRNFLILIRHRFRLT
AN7428.1  	------WKIASLSSYEDGFFKHAEFADCFTCFVDPSNYEEAPGWMLRNLLVLVKRRWGLT
          	      *.*.:* .:* ***.     * : .*.*.*.* * *.* ***:*:*:::*: * 

MG07297.1 	KANILCYRDTQARRDEPRSIVLPLASE----GPATPQTSEMPKVTGWERHPSSKLQARVI
NCU06672.1	KVQILCYRDTQPRRHEARSTILPLAMD----QVGDVELKCMPKVTGWERNGNGDLRPRVA
FG10226.1 	KTKVICYRDTWAKRHEAKSVILTIEMD----PVENLDITEMPKVTGWARSSNGKLQAQQV
AN7428.1  	KVQILRYRDGPSPRDCGRSIVVTLRLKTSQLPDGGVKDDRMPKVTGWERNPS--------
          	*.::: ***  . *.  :* ::.:  .::.      .   ******* *  .        

MG07297.1 	SLAEYMDPTRIA~DQAVDLNLKLMKWRISPKLDLEAMRSLKCLLLGAGTLGSYVSRNLM~
NCU06672.1	NLAEYMDPTRLA~DQAVDLNLKLMKWRLAPNLDLDAIKNTKCLLLGAGTLGSYVSRNLL~
FG10226.1 	NLGEYMDPARLA~DSSVDLNLKLMKWRIAPNLNLETIKNTKCLLLGAGTLGSYVSRNLM~
AN7428.1  	-----------A1DQSVDLNLKLMKWRISPNLDLEKIKGTKCLLLGAGTLGSYVARNLM0
          	           * *.:***********::*:*:*: ::. **************:***: 

MG07297.1 	GWGVRKITFVDYGNVSFSNPVRQPLFEFEDCLSGGVPKAPKAAEALKKINPGVEAEGHVL
NCU06672.1	GWGVRKITFIDYGSVSYSNPVRQPLFKFEDCHNGGKPKAIQAAEALKEIYPGVDVEGYAL
FG10226.1 	GWGVRKITFVDYGRVSFSNPVRQPLFNFNDCLEGGKPKALRAAEALKEIYPGVDSEGHAL
AN7428.1  	AWGVRKITFVDNGSVSFSNPVRQPLFNFADCLDGGAKKAYRASQALSEIYPGVESVGHVL
          	.********:* * **:*********:* ** .**  ** :*::**.:* ***:  *:.*

MG07297.1 	SVPMLGHPVLNEAQTKEDFEKLQQLIKAHDVVFLLMDTRESRWLPTVMGKAEGKIVMNAA
NCU06672.1	SVPMLDHAIHNEAKTKADFDKLKELIDSHDAIFLLMDTRESRWLPTLMGKAANKIVMNAA
FG10226.1 	SVPMLGHPFTDETKTKEDYQKLEKLINEHDAIFLLMDSRESRWLPTVMGKAAGKIVMNAA
AN7428.1  	AVPMAGHPVLDAEKTKADFEVLKGLIDAHDVIILLMDTRESRWLPTVMGKAAGKIVMNAA
          	:*** .*.. :  :** *:: *: **. **.::****:********:**** .*******

MG07297.1 	LGFDTYVVMRHGAAPKDGTESTLGCYFCNDVVAPSD0SMKDQTLDQQCTVTRPGVAAIAS
NCU06672.1	LGFDTYVVMRHGAAPNDGSEETLGCYFCNDVVVAAD0SMKDQTLDQQCTVTRPGVAAIAS
FG10226.1 	LGFDSYVVMRHGAETSPEGQTPLGCYFCNDVVAPAD0SQKDQTLDQQCTVTRPGVAPIAS
AN7428.1  	LGFDTFVVMRHGVTNNEHPEEELGCYFCNDVVAPMN0SQKDQTLDQQCTVTRPGVAAIAS
          	****::******.  .   :  **********.. : * *****************.***

MG07297.1 	AMLVEMLTSVLQHPQREHAPAPKATGPPGNPEYQRDPPDHALGIVPHQVRGFLANFQNMI
NCU06672.1	ALLVELLTSILQHPLKQHAPAPVSTGTGSAVSYERDPPDHPLGLVPHQVRGFLSNFQNMV
FG10226.1 	ALLVELLTSLLQHPLGKDAPAPQPT---SGVIPERDPPDHALGLVPHQIRGYTSTFQQIV
AN7428.1  	ALLVELLVSLLQHPLGAAAGAPQTP--------NNTQNDHPLGVIPHQIRGFLSTFENVS
          	*:***:*.*:****    * ** ..        :.   **.**::***:**: :.*::: 

MG07297.1 	I~SGESYPNCSACSSPIVGAYKSDGWEFVKKALSDKDYVLELSGLAEVQRQAEAMQNEVD
NCU06672.1	I~RGKSYPQCSACSKPILDAYKEGGWEFVKTALASRDYVAELSGLAEVQRLAEKAAAEMQ
FG10226.1 	I~RGQSYDCCSACSPKILNAYRHDGWGFVKRALQEKEYVAELSGLAEVQRRAEEMAAHVD
AN7428.1  	V0---------------------------------------------VQEMAEANLADIE
          	:                                              **. **    .::

MG07297.1 	WDEDEDVAAAEEGDGEML
NCU06672.1	WSEDEEGMDEEEGEGELI
FG10226.1 	WEEDDDLVDD--GEGELI
AN7428.1  	WDEDLEPEEITPVMD---
          	*.** :        .
```
